# Enhancing Sustainability by Improving Plant Salt Tolerance through Macro- and Micro-Algal Biostimulants

**DOI:** 10.3390/biology9090253

**Published:** 2020-08-28

**Authors:** Petronia Carillo, Loredana F. Ciarmiello, Pasqualina Woodrow, Giandomenico Corrado, Pasquale Chiaiese, Youssef Rouphael

**Affiliations:** 1 Department of Environmental, Biological and Pharmaceutical Sciences and Technologies, University of Campania “Luigi Vanvitelli”, Via Vivaldi 43, 81100 Caserta, Italy; lf.ciarmiello@gmail.com (L.F.C.); pasqualina.woodrow@unicampania.it (P.W.); 2 Department of Agricultural Sciences, University of Naples Federico II, 80055 Portici, Italy; giandomenico.corrado@unina.it (G.C.); chiaiese@unina.it (P.C.); youssef.rouphael@unina.it (Y.R.)

**Keywords:** algae, stress, salt, bioactive compounds, plant physiology, plant molecular defense

## Abstract

Algal biomass, extracts, or derivatives have long been considered a valuable material to bring benefits to humans and cultivated plants. In the last decades, it became evident that algal formulations can induce multiple effects on crops (including an increase in biomass, yield, and quality), and that algal extracts contain a series of bioactive compounds and signaling molecules, in addition to mineral and organic nutrients. The need to reduce the non-renewable chemical input in agriculture has recently prompted an increase in the use of algal extracts as a plant biostimulant, also because of their ability to promote plant growth in suboptimal conditions such as saline environments is beneficial. In this article, we discuss some research areas that are critical for the implementation in agriculture of macro- and microalgae extracts as plant biostimulants. Specifically, we provide an overview of current knowledge and achievements about extraction methods, compositions, and action mechanisms of algal extracts, focusing on salt-stress tolerance. We also outline current limitations and possible research avenues. We conclude that the comparison and the integration of knowledge on the molecular and physiological response of plants to salt and to algal extracts should also guide the extraction procedures and application methods. The effects of algal biostimulants have been mainly investigated from an applied perspective, and the exploitation of different scientific disciplines is still much needed for the development of new sustainable strategies to increase crop tolerance to salt stress.

## 1. Introduction

Algae are autotrophic photosynthetic organisms that are able to colonize even complex habitats. Algae are exposed to continuous and sometimes harsh changes in light intensity, salinity, temperature, and nutrient availability; therefore, they evolved the ability to synthesize a large array of secondary metabolites. These are mainly necessary to rapidly cope with and adapt to abiotic stress [1]. In addition to a functional diversity comprising the various adaptive traits to different environments and stresses, the algal (bio)chemical profile is also remarkably varied because of an ample taxonomic distribution and evolutionary diversity [1]. Consequently, algae extracts (AEs) contain a wide spectrum of bioactive compounds and signaling molecules, as well as macro- and micronutrients, and they have been long considered an attractive material to bring multiple direct and indirect benefits to humans and cultivated plants. 

Broadly, algae are classified in two main groups. Macroalgae (commonly named seaweeds) are multicellular, marine, or fresh-water organisms, frequently separated in three divisions: brown (phylum Ochrophyta, class Phaeophyceae), red (phylum Rhodophyta), and green (phylum Charophyta and phylum Chlorophyta) algae [2]. Macroalgae such as *Undaria* and *Laminaria* are harvested in coastal areas for food, folk medicine and, more recently, the production of nutraceuticals and cosmetics [3]. The second group is represented by microalgae, which comprise blue and green algae. Microalgae are evolutionarily diverse photosynthetically unicellular organisms, virtually present in any aquatic eco-system and top-soil. Among them, *Spirulina* spp. and *Chlorella* spp. have a large economic value for the production of nutraceuticals and prebiotics, while *Dunaliella* spp. and *Haemotococcus pluvialis* are used for the synthesis of common anti-oxidants (beta-carotene and astaxanthin) [4,5].

The use of algae biomass as a fertilizer is an ancient agricultural technique, known since Roman times and implemented for instance, in Britain, France, Spain, and especially in East Asia (e.g., China and Japan) [6]. AEs or algal derivatives (ADs) have been typically applied to soil as organic, slow-release fertilizer, and soil amendment to return nutrients (both carbon and macro-elements) and improve soil physical and biological properties. More recently, AEs and ADs are causing interest as plant biostimulants (PBs), with the main goal of increasing Resource Use Efficiency (RUE), abiotic stress response, and resilience to water deficit in plants [7,8,9,10,11,12,13,14,15,16,17,18]. Moreover, the deployment of AEs as PBs is expected to help sustainable agricultural intensification also by improving the uptake of non-renewable chemical fertilizers [8,9,11]. 

AEs have an unexplored potential as PB for salinity, which is still one of the most harmful abiotic stresses in agriculture, for different reasons. They include the AEs’ chemical and biochemical diversity, the range of biological effects on crops, and the shared molecular pathways between algae and higher plants. The effectiveness of PBs to alleviate the threat of salinity depends also on the timing of application. In particular, applications can be carried out at three different stages: before the stress affects the cultivation (priming), during stress conditions (defense), and after (recovery), when plants already display evident signs of stress. Although a clear-cut distinction based on the effects on the leaf physiological parameters is rarely present, biostimulants for defense and recovery should be enriched in compounds that rapidly act as osmoprotectants (e.g., proline, amides, γ-aminobutyric acid (GABA), glycine betaine). For priming, it would be necessary to exploit AEs rich in signaling molecules that are able to activate the endogenous plant biosynthesis of protective metabolites and the related physiological adaptations to stress. The application timing should be also assessed on a crop-by-crop base, as it is expected to differ from species to species, phenological stages, salt concentrations, time of exposure, and management practices [19]. For all these reasons, the identification of the proper time of biostimulant application, alongside with extract type and dose, is important to avoid product loss and unanticipated results [20]. Moreover, another area of interest for unleashing the market potential of AEs and ADs is related to the reduction of the variability in product quality and composition, which often results in an unreliable performance of the commercial products. The vast chemical diversity in algae implies that the extraction technique (i.e., the solid or liquid separation of biostimulating fractions by using standardized procedures) is an important factor for the application of AEs in agriculture, and its improvement requires advances from process engineering, phyto- and analytical chemistry. Moreover, it is necessary to increase our knowledge on the bio-molecular action mechanisms. Unraveling the molecular effects of these diverse metabolites or metabolite mixtures is a complex task that justifies a multi-disciplinary approach embracing genomics, transcriptomics, proteomics, and metabolomics, as well as plant physiology. New exciting biological advances are expected from the application of molecular genetics to investigate the unexplored plant responses to algal extracts. This information is also valuable to ensure the recovery of the appropriate fractions or classes of compounds from algae and to guide the selection of the appropriate application scheme to each crop.

The aim for this review is to offer a critical discussion of the extraction techniques and the chemical composition of seaweed- and microalgae-based extracts and biostimulants. Moreover, we provide an update of the possible physiological, biochemical, and molecular mechanisms of the interaction between AEs and plants in relation to salt stress. All this knowledge will ultimately increase our chances to sustainably secure yield stability under salt stress conditions using algae extracts.

## 2. Extraction Techniques, Chemical Characteristics of Macroalgae and Microalgae

The commercial production of AEs requires easily accessible and affordable raw material and has been traditionally based on the management of renewable natural resources. Production methods of algal biomass for metabolites’ extraction currently range from ocean farming to photobioreactors, including a series of medium-level technological options, such the open pond systems [21]. Although the energy requirement, conformance to specifications, reconfigurability, and scalability of the various systems are very different, there is a consensus that algal cultivation is economically viable mainly to manufacture high-value chemicals [22], as also indicated by the commercially available products [23]. Many studies have addressed the economic feasibility of microalgae as a source of molecules for the health sectors, including cosmetics, while less emphasis has been dedicated to the environmental protection sector [24]. Different factors imply that also manufacturing algal-based biostimulants has appealing possibilities. Firstly, compared to the isolation of a specific metabolite, the use of the entire algal biomass or crude extract as PB, an advantageous strategy for agriculture, increases the economic feasibility [25]. Moreover, algal-based biostimulants provide economic benefits when used in quantities that are a fraction of that required as algae feed, manure, or fertilizer. Furthermore, the components of interest for plant biostimulation are varied (as detailed in Section 3) and go well beyond those of interest for producing pharmaceuticals, functional foods, cosmetics, dietary supplements, and industrial pigments. Therefore, the feasibility of AEs in agriculture could be easily increased by an integrated co-production strategy [12]. This includes the cultivation of algae in on-farm low-tech unstirred ponds, as well as the exploitation of other activities, such as wastewater treatment and atmospheric CO_2_ mitigation technologies [26]. Finally, moving beyond profit, the growing need for a more resource-efficient and sustainable bioeconomy prompts the use of renewable biological resources for industrial purposes and requests a better integration of agriculture with blue biotechnology [27].

As a result of their high growth rate and abundance in coastline marine habitats of various countries, brown algae, typically kelps (Laminariales), have been long used in agriculture. Conversely, in addition to ecological and sustainability concerns that may arise in case of an excessive exploitation, the use of natural resources, harvested for instance, in different seasons and years, is considered one of the factors that hinders the development of tight technical operational standards and of a constant product quality. The most common algae for the production of commercial plant biofertilizers and biostimulants are brown algae such as *Ascophyllum nodosum*, *Ecklonia* spp., *Fucus* spp., *Laminaria* spp., and *Sargassum* spp. [28,29,30,31,32]. Other common species include *Fucus serratus*, *Ulva lactuca,* and *Kappaphycus alvarezii* [33,34,35,36,37,38]. The majority of commercially available algal-based biostimulants are aqueous extracts. They range in color (from almost colorless to an intense dark brownish-black), odor, viscosity, and composition [13]. It is difficult to provide key procedures and guidelines because AEs are not a homogenous class of products, and companies do not disclose methods and conditions used for the preparation of commercial products. Usually, the liquid extraction is performed using water, alkalis, or acids, also in combination with a physically disruption of the algae by low-temperature milling, high-pressure homogenization [39], ultrasonication [40], pulsed electric fields [40,41,42], or bead milling [42,43,44].

Since extraction methods must be targeted to the different classes of metabolites, it is unreasonable to expect that one extraction method is suitable for all metabolites. For instance, algal proteins are usually extracted by means of aqueous, acidic, and alkaline methods, and collected by centrifugation and recovery using techniques such as ultrafiltration, precipitation, or chromatography [45]. In particular, a two-phase acid and alkali treatment efficiently extracted proteins from *A. nodosum*, *L. digitata*, and *Ulva* spp. [46]. Physical grinding by a homogenizer followed by immersion in ultra-pure water was employed for *Sargassum* spp. and *Ulva* spp. [47]. Osmotic stress or shock have been reported to enhance the yield of protein extraction in *Palmaria palmata* [48]. In order to increase the protein extraction efficiency, polysaccharidases (starch, alginates, cellulose, fucoidans, galactans, laminarin, and xylans) that degrade enzymes can be applied before protein extraction for improving the breakdown of the algal cell wall [47]. These pre-extraction phases certainly increase yield, but with the risk that conventional chemical, mechanical, and enzymatic methods could release proteases from vacuoles, damaging the integrity of extracted algal proteins [38]. However, Suarez Garcia, et al. [49] demonstrated that the yield of functional protein in AEs could be significantly increased, up to 50.4% dry weight (dw) of proteins, by applying a cell disintegration through a single bead-milling step for short times under mild conditions (e.g., room temperature, pH 6.5, and without the addition of chemicals). This technique allowed lower energy consumption than other disintegration methods, such as ultrasonication and electric fields. Ursu et al. [50] demonstrated that through using high-pressure (2.7 kbar) cell disruption at pH 12.0 or 7.0, it was possible to obtain a protein yield of 98% and 71%, respectively. On the other hand, the neutral pH allowed to yield proteins with a higher emulsifying capacity [50].

Novel methods that have been used for the extraction of proteins as well as other metabolites include ultrasound-assisted extraction (UAE), pulsed electric field (PEF), and microwave-assisted extraction (MAE) [45]. The radical sonochemistry of UAE produces the formation, growth, and implosion of bubbles by acoustic cavitation that highly increase the chemical excitation of the sonicated liquid and its contents, causing the breakdown and degradation of the targeted structures and compound. This method reduces the processing time, the use of solvents, and the energy requirement, while increasing the yield and purity of the final product. For instance, UAE enhances protein extraction from *A. nodosum* by 540% and 27% when applied with acid and alkaline treatments, respectively, reducing the processing time from one hour to 10 min [46]. PEF is a non-thermal technique based on short, high-voltage pulses. In microalgae, it was initially used to extract lipids for biofuel production, but it is now also applied for extracting other metabolites, such as carbohydrates, carotenoids, and chlorophyll [41,51]. It has been reported that PEF increase the protein yield from *Chlorella* spp. and *Spirulina* spp. by 27% and 13%, respectively [52]. However, Lam et al. [53] demonstrated that in species such as *Chlorella vulgaris* and *Neochloris oleoabundans*, which have a thick cell wall, PEF treatments, even under severe energy inputs (10 to 100 times higher than bead milling), can release only about 27% of the proteins released by the bead milling technique. This technique was efficient in releasing ions [53]. Postma et al. [42] showed that in *Chlorella vulgaris*, carbohydrates can be selectively released by PEF combined with temperature treatment. 

MAE is a relatively new technique based on irradiation with microwave energy to help the transfer of the compounds of interest into a chosen solvent. Essentially, it consists of heating the algae, causing the formation of bubbles that under high pressure can cause the breakdown of algal cells with the release of the internal compounds [40]. MAE cannot be used with dried or lyophilized algae biomass [54]. Over the last decade, subcritical water extraction (SWE) and supercritical fluid extraction (SFE) have become also popular [55]. SWE uses hot water (higher than 100 °C) under high pressure (approximately 10 bar) to maintain water in a liquid state. It offers the possibility to replace organic solvents with the highly affordable, non-toxic, and safe water [55]. For instance, SWE was used to enhance the extraction of proteins and carbohydrates in *C. vulgaris* [56]. SFE uses temperature and pressure above the critical point of a substance, usually employing carbon dioxide as solvent. It these conditions, the supercritical fluid maintains the density of a fluid but the viscosity of a gas [57]. SFE is a relatively rapid method, whose selectivity can be tailored according to the extraction conditions. SFE is also considered a green method because it does not require high amounts of solvents, but it requires specific equipment and so far, it has been utilized mainly for lipid extraction [45].

Membrane-based technologies (e.g., microfiltration, ultrafiltration, nanofiltration, and reverse osmosis) are non-thermal and eco-friendly methods usually applied in combination and in conjunction with a cell disruption technique. They are based on semi-permeable membranes to separate certain compounds from liquids according to specific parameters such as the molecular weight [40]. Microfiltration is useful for removing algae cell wall components and bacteria with a molecular weight higher than 200 kDa. For instance, it has been used to recover about 70–90% algal biomass from wastewater treatments [58]. Ultrafiltration can help isolate metabolites between 1 and 200 kDa; nanofiltration can remove monovalent ions to reduce osmotic pressure, while reverse osmosis can decrease the liquid volume [59]. Ultrafiltration has been already used together with CO_2_–SFE extraction, cell homogenization, or ultrasound for polysaccharides or protein enrichment [45]. 

## 3. Main Bioactive Compounds of Macroalgae and Microalgae that Affect Growth and Salt Tolerance 

The biostimulatory action of AEs on the growth, yield, and resilience of crop plants was initially attributed to the supply of essential nutrients and to the improvement of soil texture, structure, and water retention capacity. It is well established that AEs contain high amounts of macronutrients, especially N, P, and K [14,60]. For instance, *Arthrospira* spp. contain 6.7%, 2.5%, and 1.1% on a dry basis of N, P, and K, respectively [61]. Another applied advantage of using AEs is that they are able to keep minerals in a soluble form [13]. 

The use of AEs at relatively low doses (e.g., as plant biostimulants) has shifted attention to components other than inorganic nutrients. Several studies report that AEs can promote plant growth because of a mixture of signaling molecules and metabolites such as phenolics, phytohormones, carbohydrates, betaines, amino acids, carotenoids, vitamins, and polyamines [1,15,62,63] (Table 1). As a result of their direct and well-known role in plant response to abiotic stress, in this review, we mainly focus on phenolics and phytohormones.

It has been long known that marine algae can have a high concentration of phenolics. These compounds usually accumulate under stress and have the ability to scavenge Reactive Oxygen Species (ROS), to chelate metal ions, and to stabilize membranes and proteins [64,65]. Compared to terrestrial plants, the phenolics from marine macroalgae are more variable, ranging from simple molecules such as phenolic acids to highly complex and specialized compounds. For instance, brown algae typically possess phlorotannins, a type of polyphenols absent in terrestrial plants that is involved in the response to both biotic and abiotic stress [66]. Brown seaweeds such as *A. nodosum, F. vesiculosus,* and *F. serratus* are rich not only in phlorotannins but also in phloroglucinol, eckol, and dieckol. These compounds have more phenolic rings than other phenolics and, therefore, they are expected to detoxicate ROS more efficiently [65,67,68]. *Ankistrodesmus* spp., *Spirogyra* spp., *Euglena cantabrica,* and *Caespitella pascheri* are rich in gallic, syringic, protocatechuic, and chlorogenic acids, along with flavonoids, such as catechin and epicatechin [69]. *Nannochloris* spp., *Tetraselmis suicica*, *Nannochloropsis gaditana,* and especially *Phaedactylium tricornutum* are rich in phenolics, such as the protocatechuic acid, and carotenoids, such as E-fucoxanthin [70]. 

Phytohormones, in particular auxins and cytokinins, are also considered important factors to enhance plant growth, yield, and defense response, especially against abiotic stress [71]. Auxins and cytokinins were detected and quantified in 24 microalgae strains from four different families of green algae (*Chlorophyceae*, *Trebouxiophyceae*, *Ulvophyceae,* and *Charophyceae*) [63]. Auxins concentration was 2- to 4-fold higher than that of cytokinins in 15 out of the analyzed 24 strains [63]. Indole-3-acetic acid (IAA) and indole-3-acetamide (IAM) were the only detected auxins [63]. Their concentration greatly varied, with IAA ranging from 0.09 to 12.52 µg g^−1^ dw, and IAM ranging from 0.03 to 17.39 µg g^−1^ dw. IAA and IAM are present in several red algae (Rhodophyceae) species collected from the Brazilian coast [72]. IAA has been also found in the macroalgae *Kappaphycus alvarezii* and *Sargassum tenerrimum* [34], while IAA and indole-3-butyric acid (IBA) have been identified in wild *Ulva* species [34]. In the same study relative to 24 microalgae strains, 19 cytokinins were also identified [63]. Total cytokinin concentrations ranged between 0.06 and 4.61 µg g^−1^ dw, with *cis*-zeatins being the main type. The profile of 31 seaweeds (5 Chlorophyceae, 7 Phaeophyceae, and 19 Rhodophyceae) collected in South Africa indicated the presence of 13 isoprenoid cytokinin conjugates with higher concentrations of isopentenyladenine and *cis*-zeatin conjugates. A study of 11 Rhodophyceae species identified 11 isoprenoid cytokinin conjugates, with *cis*-zeatin conjugates being the most abundant [38]. Regarding other phytohormones, gibberellins and brassinosteroids have been identified in algae for the first time in 2013. Around 20 different gibberellins were identified in strains of *Chlorophyceae*, *Trebouxiophyceae*, *Ulvophyceae,* and *Charophyceae*, with concentrations ranging from 0.34 to 4.75 µg g^−1^ dw, with GA_6_ representing the most abundant active gibberellin [62]. Brassinolide and castasterone were the most relevant brassinosteroids, whose concentration ranged from 0.11 to 0.98 µg g^−1^ dw [62]. Brassinosterioid biosynthesis is associated to stress also in algae. Under salinity (36 g L^−1^ NaCl), six microalgae species (*Chlorococcum ellipsoideum*, *Gyoerffyana humicola*, *Nautococcus mamillatus*, *Acutodesmus acuminatus*, *Protococcus viridis,* and *Chlorella vulgaris*) rapidly accumulate brassinosteroids, in particular, castasterone, and in lower amount brassinolide, homocastasterone, and typhasterol. Finally, abscisic acid (ABA) was first detected in all 64 algal species originating from 9 divisions, 20 classes, and 36 orders [49], leading to the proposition that this hormone is universally distributed within the algal kingdom. However, the role of ABA in algae is still not well characterized, with some evidence indicating an involvement in stress responses such as desiccation and salinity [73,74]. 

The concept that endogenous algal hormones are a main component of the biostimulatory effects of AEs is supported by the lack of a strong linear relation between seed germination and dose. For instance, a high dose of biostimulants can cause the inhibition of seed germination [75], an effect that cannot be easily explained with a nutritional or manurial function of the algal extract. On the other hand, the role of the algal hormones and of low molecular weight organic compounds has been also questioned. For instance, it has beendiscussed that the concentration of phytohormones in commercial AEs is not fully adequate to cause major physiological responses [18]. 

More attention has been given to larger molecules such as oligomers and polysaccharide elicitors. Both macro and microalge synthetize polysaccharides (e.g., agars, alginates, carrageenans, and fucans) that are not present in land plants [15,76]. These polymers, and more often their degradation products, can act as elicitors that show a variety of biological activities when applied to plants, which are mainly related to the activation of hormonal stress signals such as salicylic acid (SA), jasmonic acid (JA) and thylene, and an increased resistance to biotic stress such as pathogens, insects, and viruses [77,78].

Algae are rich in proteins and derivatives that are not frequent in land plants. For instance, typical proteins include phycobiliproteins, agglutinin glycoproteins, and mycin-binding agglutinins. Endogenous bioactive peptides comprise carnosine and glutathione. Some algae strains contain uncommon amino acids or amino-like compounds, such as D-homocysteic acid, γ-aminobutyric acid (GABA), ornithine, citrulline, hydroxyproline, taurine, or kainic acid [79,80]. Taurine is present at high concentration (up to 4 g/16 g N) in some red algae, such as *Porphyra* spp. from Korea and Japan, while phosphoserine is usually present at high concentrations in brown algae [81]. Taurine and phosphoserine can act as antioxidants. In particular, phosphoserine-containing peptides can lower lipid peroxidation and increase intracellular glutathione and the expression of anti-oxidant enzymes [82,83]. As it occurs also in land plants, proline can act as an osmolyte and cellular protectant and accumulate in algae under salinity stress (e.g., in the green algae *Stichococcus bacillaris*) [84]. 

Considerable amounts of polyamines, in particular putrescine and spermidine, were also found in unicellular and multicellular green and red algae (Chlorophyta, Charophyta, and Rhodophyta) [85,86]. Polyamines, aliphatic compounds deriving from two or more amino acids, play multiple roles—for instance, in the regulation of the cell cycle, and in sugar and lipid homeostasis. Polyamines are also important in plants for their antioxidant capacity and role in osmotic regulation both as osmoprotectants and signaling molecules [86]. Trehalose, a non-reducing disaccharide that act also as an osmolyte, carbon reserve, and stress protectant [87], is accumulated in algae such as *Chlamydomonas*, *Chlorella,* and *Scytonema* under salinity [88]. Polyols, such as the important osmoprotectans sorbitol and mannitol, were detected in species such as *Platymonas suecica*, *Stichococcus chloranthus,* and *Stichococcus bacillaris* under salinity [88]. Betaines (in particular glycine betaine, γ-aminobutyric acid betaine, and proline betaine), tertiary sulfonium analogues (partucularly 3-dimethyl-sulphoniopropionate), or a mixture of the two types of compounds are widespread in marine algae [21]. In *Chaetomorpha capillaris*, glycine betaine constitutes about 2% of dw, while in other algae species, the major betaine or tertiary sulfonium metabolites account for less than 1% of dw and frequently less than 0.1% [32]. The main betaine in *A. nodosum* is γ-aminobutyric acid betaine, which represents 0.02–0.07% of the dw [33], with glycine betaine, δ-aminovaleric acid betaine, and laminine present in lower amounts [28]. Betaines may function as osmolytes or ROS scavengers, and they can interact with molecules and membranes, preserving their structure and activity [89]. In *Nannochloris* spp., betaines account for 0.023% of dw, while protein hydrolysates, proline, carbohydrates, and cytokinins and cytokinin-like metabolites account for 88.0%, 5.7%, 9.4%, and 0.012%, respectively [64]. MacKinnon et al. [28] reported the same amount of betaines in *A. nodosum*-derived commercial AEs, while Craigie [13] described the same concentration of cytokinins in commercial AEs. 

Finally, free amino acids in marine algae include also low molecular weight soluble acid-like compounds such as mycosporine-like amino acids [90]. Thanks to their conjugated double bonds that absorb from 310 nm (UV-B) to 360 nm (UV-A), these compounds are mainly involved in protection from UV radiation and oxidative damage, but they also act as osmolytes to protect cells against salinity, desiccation, and cold stress [90].

It is undeniable that an important step in the characterization of an algal extract is the establishment of the chemical identity of the product. This information is also useful to biologically evaluate, and possibly explain, the biostimulatory effect. Nonetheless, the various active fractions described in the literature, which are summarized in Table 1, also imply that the AEs effect on plants under salinity very likely results from the presence of multiple, chemically unrelated compounds.

## 4. Mechanisms of Salinity Stress Tolerance in Macro and Microalgae-Treated Plants

Salinity negatively affects plants’ growth, development, and yield via pleiotropic mechanisms involving osmotic stress, nutrient imbalance, ion toxicity, and oxidative stress [19,99,100,101,102]. When the soil electrical conductivity (EC) approaches the threshold limit of 4 dS m^−1^, corresponding to about 40 mM of sodium chloride, the majority of the glycophytes display a reduced capacity to uptake water from soil [103]. In addition, salinity impairs transpiration and stomatal conductance, affecting the expansion of older leaves and the emergence of new leaves and lateral buds [104]. In case of a prolonged saline stress, sodium accumulates in plant cells, exerting nutritional imbalance, as well as osmotic and ion-specific effects. An excessive cellular concentration of sodium can disturb the synthesis and activity of enzymes, the stability and structure of membranes, and the electron transport chains [19,104]. Sodium may substitute potassium in key enzymatic reactions, impairing protein synthesis and the photosynthetic machinery [99,100,105]. Plants try to cope with salt stress, eliciting several defense responses whose overall effect is to reactivate enzymatic systems, physiological functions, and growth [106]. AEs can boost plant defense systems, acting both on plant RUE and the synthesis of protective compounds facilitating the osmoregulation and antioxidant response, protecting cells and subcellular structures. Recent studies highlight that AEs can elicit different biochemical, physiological, and molecular mechanisms to enhance plant tolerance to salinity [60,107]. In particular, they are able to mitigate the effects of salt stress inducing protective metabolites and/or the activation of metabolic pathways, which not only contribute to enhance plant growth and yield but also increase quality [11,108,109] (Table 2 and Table 3).

### 4.1. Biochemical and Physiological Mechanisms in AE-Treated Plants

Brown seaweeds are the most employed algae for the industrial sector, mainly because of their large size and availability throughout the year. Among them, *A. nodosum* is probably the most common species. Although its biomass has been long used as fertilizer, studies carried out with commercial formulations containing *A. nodosum* extracts proved that they act as enhancers of growth, yield, quality, and adaptation to stress in salinized lettuce (*Lactuca sativa*), thale cress (*Arabidopsis thaliana)*, tomato (*Solanum lycopersicum*), passion fruit (*Passiflora edulis*), and avocado (*Persea americana*) [108,114,116,117,131,132,133,134]. Several studies have been carried out in soil, thus making it difficult to entangle the direct (e.g., nutritional and biostimulatory activities) and indirect (e.g., manurial effect) impact on crops. Moreover, the diversity of potentially active compounds in AEs does not make it easy to pinpoint a single (bio)chemical class that is solely responsible for the effects on plant physiology. 

Protein hydrolysates have been included among the active ingredients of PBs that are able to promote plant growth, vigor, and yield even under suboptimal conditions through a coordinated regulation of C and N metabolism that enhances RUE [7,8,9]. In addition, also amino acids, present in algae or derived by algae proteins, can promote plant growth and crop yield and contribute to abiotic stress tolerance in lettuce [135]. In *A. nodosum* commercial extracts, there are high concentrations of proline, GABA, and branched chain amino acids (BCAAs), in addition to betaines and polyols (mannitol and sorbitol) [108]. These AEs significantly enhanced tomato plants’ growth and yield under salt treatment, and they ameliorated RUE and stress tolerance, allowing the accumulation of minerals, antioxidants, and essential amino acids in fruits, leading also to an overall improvement of their nutritional value [108]. Proline in plants can act not only as osmolyte, balancing the osmotic pressure of cell cytosol under salinity, but it can also stabilize the structure of membranes and proteins, scavenging ROS and buffering cellular redox potential. Moreover, proline induces the expression of genes with proline responsive elements (e.g., PRE, ACTCAT) in their promoters, which is essential for salt stress response [80]. GABA as a zwitterion also behaves as a non-toxic osmolyte, balancing the decrease in water potential during cellular dehydration caused by salinity and functioning as an antioxidant for the stabilization and protection of thylakoids and macromolecules in plants [80]. Both GABA and proline accumulate in algae and plants in response to salinity and other abiotic stresses [101,136,137]. In addition, these two osmolytes can be also rapidly metabolized after relief from stress, eventually providing energy, carbon, and nitrogen useful also to recover and repair salt stress-induced damages [80,138]. 

It is interesting that AE treatment under moderate drought enhanced *Spiraea* and *Pittosporum* plant water status and gas exchange parameters and it was associated with a reduction of proline in leaves, implying that AE-treated plants sense a less stressful environment [139]. However, under low and high salinity conditions, proline accumulated in higher quantities in AEs-treated *Asparagus aethiopicus* [113], suggesting that the interaction plant x AE x stress goes beyond a direct, linear compound-specific elicitation. BCAAs may also play a role as osmolytes to counteract the effects of water stress and as alternative electron donors for the mitochondrial electron transport chain [140]. Moreover, these amino acids can scavenge ROS through a not fully elucidated mechanism [101,141]. *A. nodosum* extracts boost RUE in avocado under salinity, enhancing growth and yield [117]. The extract of this alga helps plants to better discriminate between sodium and potassium, maintaining higher K/Na ratios [115]. *A. nodosum* extracts increase the content of phenolics, antioxidant activities, chlorophyll, sugars, and proline content in *Asparagus aethiopicus*, enhancing transpiration and photosynthetic rates as well as stomatal conductance [113]. The treatment with acidic polysaccharides extracted from the brown seaweed *Lessonia nigrescens* enhanced the antioxidant activities and ions efflux (or compartmentalization), decreased lipid peroxidation, and improved salinity tolerance and plant growth in Triticum aestivum wheat seedlings under salinity [109]. The application of *Dunaliella salina* sulphated exopolysaccharides to tomato plants activated antioxidant activities and increased shoot and root lengths and dry and fresh matters [120]. An extract from the red alga *Kappaphycus alverezii* was supplied to three commercial durum wheat varieties under salinity and drought stress during both vegetative and reproductive growth phases [36]. These extracts contain high amounts of carrageenan, a seaweed phycocolloid, and plant growth regulators, such as IAA, kinetin, trans-zeatin, and GA3. Their application reduced the ionic imbalance and ROS concentration by increasing the potassium to sodium ratio (K/Na) as well as the content of calcium osmoprotectants, proline, total protein, and free amino acids. These consequently increased shoot and root length and weight, total chlorophyll, and carotenoids, while decreased electrolyte leakage and lipid peroxidation in durum wheat [36]. The regular and constant application of seaweed extract and humic acid on creeping bentgrass increased the superoxide dismutase (SOD) activity (47% and 181%) together with photosynthesis improvement [110]. This effect on SOD activity could be explained by the AE hormonal and osmolyte content. Similar results were observed on a *Paspalum vaginatum* cultivar when a foliar application of *A. nodosum* extract was regularly applied in presence of salt or drought stress [115], with an enhanced antioxidant defense mechanism and a significant increase of SOD, catalase (CAT), and ascorbate peroxidase (APX) activities in AE-treated plants. An *A. nodosum* extract to eggplants under salinity increased phenols, tannins, and total soluble sugars content, as well as SOD and APX enzymatic activities, promoting a higher potassium to sodium ratio [91]. The application of *Ulva lactuca* extracts to *Triticum aestivum* seedlings under salinity enhanced plant fresh and dry weigh, APX, and glutathione reductase (GR) activities at a 1% application rate. At 10%, it increased SOD and CAT activities [35]. A significant increase of activities of SOD, APX, CAT, and POD was also found in wheat plants (*T. aestivum* L., cv. Giza 168) irrigated with 10% or 20% seawater and treated with aqueous AEs of *Scenedesmus obliquus* and *Spirulina platensis* [128]. This was accompanied by an increase of photosynthetic pigments (chlorophylls and carotenoids) and glutathione, with a decrease of lipid peroxidation. A similar increase of SOD APX, and CAT was observed in *Triticum durum* under 0, 2, and 4 g L^−1^ of NaCl and treated with aqueous AE of *Ulva rigida* and *Fucus spiralis*, respectively [124,130]. 

*Dunaliella salina* and *Phaeodactylum tricornutum* AEs reduced superoxide radical production and lipid peroxidation, with a general increase of germination rate and root growth in bell pepper [122]. Similarly, four out five of marine algae (*Laurencia obtuse* > *Sargassum muticum* > *Padina pavonica* > *Galaxaura obtusata*) crude extracts were able to regulate sodium uptake, as well as increase CAT and SOD activities, carbohydrates, and shoot and root length in *Triticum vulgare* seedlings [125].

Overall, a whole range of plant responses has been described at the biochemical and physiological level in relation to the use of algal biostimulants under salinity. The increased plant tolerance to salt is likely to result from a cumulative effect of different components. Moreover, the effects of AEs biostimulation overlap with salt-stress response and more generally with the adaptive mechanisms to abiotic stress. The plant response to algal biostimulation is comparatively much less detailed than salt-stress response, yet it is notable that this commonality is broad, referring for instance to mechanisms related to ion homeostasis, ion exclusion, biosynthesis of osmoprotectants, and increases of antioxidant compounds, enzymatic activities, and phytohormones playing important roles in plant response to environmental stress. 

### 4.2. Molecular Response in Plants to AEs Treatment

It is not surprising that considering the complex chemical and biochemical nature of the AEs, transcriptomics typically indicate that several plant genes and pathways are affected by algal biostimulation. As a result of the wealth of genomic information and genetic resource, studies on the plant molecular response to AEs has been also performed on the non-crop model plant *Arabidopsis thaliana*. *A. nodosum* extract was able to induce strong transcriptomics changes in *Arabidopsis*, upregulating 257 genes while downregulating 262 genes after 5 days of salinity stress [131]. The treatment of *Arabidopsis* under salinity with a neutral and an alkaline *A. nodosum* AE prepared at high temperature (>100 °C) also determined strong changes of the transcripts profile [112]. In particular, both studies demonstrated that in *Arabidopsis*, *A. nodosum* extracts can induce a stress response mediated by late embryogenesis-abundant (LEA) proteins and dehydrins, which are able to act as hydration buffers, binding large numbers of water molecules, ion trappers, antioxidants, and stabilizers of membrane and protein structures protection [144,145]. 

The weekly application of an *A. nodosum* extract on asparagus grown under salt stress (2000 and 4000 ppm NaCl) upregulated specific stress-related genes, particularly *ANN1*, *ANN2*, and *PIP1*, as well as aquaporin and water stress-related genes such as *P5CS1* and *CHS*, which are involved in the synthesis of proline and chalcone, respectively. Chalcone is the precursor of the flavonoid/isoflavonoid biosynthesis pathway [146]. Algal treatments increased salinity tolerance by enhancing proline and phenols content, along with antioxidant activities in *Asparagus aethiopicus* [113]. *A. nodosum* extracts enhanced cytokinins and ABA biosynthetic genes, while auxin biosynthetic genes were repressed in *Arabidopsis* [18]. In the same plant species, an ethyl acetate fraction of *A. nodosum* extract enhanced the transcription of *SnRK2* kinases. These proteins, by phosphorylating ABA response element-binding factors, are important regulators of plant response to abiotic stresses and ABA-dependent plant development [147,148]. Extracts from the same algae are also involved in the post-transcriptional and post-translational regulation of stress-responsive Transcription Factors (TFs). Zinc Finger, MYB, and AP2 TFs are significantly upregulated by AEs in *Arabidopsis* [131]. Other TFs transcriptionally activated by AEs are DRE-binding protein (DREB1A)/CRT/DRE-binding factor 3 (CBF3) and DRE-binding protein (DREB1C)/CRT/DRE-binding factor 2 (CBF2), COR47, NF-YA, COR15A, AGF2, CCA1, and LHY1, which enhance stress tolerance in plants [112,131,132]. Particularly, DREB2A and DREB2B transactivate the DRE cis element of osmotic stress genes and thereby are involved in maintaining cell osmotic balance [131,149]. *A. nodosum* extract increased the activity of antioxidant enzymes, which are able to scavenge ROS and reduce lipid peroxidation in turf grass under salinity [115], and it reduced oxidative damage by enhancing the expression of glutathione S-transferase in *Arabidopsis* under salinity [131]. Probably both actions may depend on the modulation of the expression of miR398 [132], which is an miRNA that is suggested to be directly linked to plant stress responses to oxidative stress, water deficit, salt stress and ABA [150]. Moreover, the modulation of expression of miRNA399, miR827, and miR2111b for P uptake and miR395 for sulfur uptake and transportation can enhance these nutrients’ use efficiency in plants under salinity [132,134]. *A. nodosum* AE was able to determine the overexpression of genes related to stress in soybean, among which *GmCYP707A1a* and *GmCYP707A3b* were identified as ABA 8′-hydroxylases involved in ABA catabolism [31]. *A. nodosum* extracts also affect the expression of genes in *Arabidopsis* involved in the biosynthesis of carbohydrates (starch, sucrose, raffinose), amino acids (proline, isoleucine), and sugar alcohols (inositol, trehalose), as well as the biosynthesis and transportation of flavonoids under salinity [148]. Exopolysaccharides extracted from *Dunaliella salina* activated antioxidant defense and several metabolic mechanisms related to the jasmonic acid pathway in tomato [120]. Polysaccharides from *Lessonia nigrescens* were able to induce the expression of *SOS1* and *NHX2* genes, which are involved in the efflux and compartmentalization of Na^+^, thus attenuating salt stress damage [109]. Polysaccharides contained in microalgae, such as β-glucan, have also been suggested to boost plant growth thanks to their interaction with leucine-rich repeat membrane receptors involved in the regulation of cell expansion-associated gene expression [60]. Carrageenan and plant growth regulators (e.g., IAA, kinetin, trans-zeatin, and GA3) contained in the red alga *Kappaphycus alverezii* increased durum wheat tolerance to salt and drought stresses by enhancing the expression of stress-responsive wheat MAP kinase, WRKY transcription factor, and antioxidative genes [36].

The examples provided in this paragraph underline the complexity of the plant response to AEs. As also evident for the biochemical and physiological plant response, current evidence supports a significant molecular connection between the improved plant performance and the activation of genes involved in defense pathways. Nonetheless, omics studies are still in their infancy, and an integrative genetics and genomics knowledge is still missing. This is also due to the lack of a shared model system for basic research and the applied nature of several studies. A further development will be the use of multi-omics approaches because they can provide the information necessary to unravel the functional consequences of the AEs use in agriculture considering different genetic, environmental, and agronomic management factors and their interactions.

## 5. Conclusions

The worldwide increasing salinization of arable land renders salinity one of the most harmful threats to modern agriculture. AEs from brown, green, and red macroalgae as well as from microalgae represent an additional option to improve defense responses in crops under salt-stress conditions. Omics, in particular transcriptomics, have allowed advancements on the physiological and molecular mechanisms lying beneath the AEs effects. However, there are various issues that still need to be addressed for realizing the full potential of AEs under optimal and suboptimal (salinity) conditions. For instance, the high variation in product quality still leads to difficulty in standardization and unreliable performance in professional agriculture. Moreover, a critical factor is the inadequate knowledge on the exact crop stage that will benefit from biostimulation (e.g., before, during or after the stress occurs). Similarly, it is also necessary to improve our knowledge regarding the optimal mode and rate of application as well as the impact of climatic (e.g., relative humidity, air temperature, and global radiation) and morpho-anatomical factors (e.g., leaf permeability) to get maximum benefits for crops. 

In the coming years, researchers, the biostimulant industry, and extension specialists need to collaborate to develop a second generation of AEs products (Algae 2.0) with more wide-ranging, crop-tailored, and consistent biostimulation action. To address all these issues, it will be worth exploring the additive or synergistic effects of seaweeds (brown, red, and green) and microalgae (green and blue-green algae), in order to provide crude extracts suitable for a range of conditions, stresses, crops, and application methods. Moreover, it will be necessary to clarify at the molecular level the effect of specific algal extracts, compounds, or mixtures on plant physiology and phenology. This will allow the exploitation of the biostimulatory functional diversity in algae through the extraction of specific fractions or definite chemical classes, going beyond the random sampling of algal diversity. We have now a good understanding of the plant molecular tolerance mechanisms to salt, and this knowledge should be exploited to better evaluate and categorize AEs. This route will ultimately allow identifying specific active fractions from AEs that are central for the biostimulant industry to match the ever-growing demand for the sustainability, profitability, and safety of primary production.

## Figures and Tables

**Table 1:** A summary of representative bioactive compounds in algal extracts and their effects on plants under salinity. GABA: γ-aminobutyric acid, IAA: indole-3-acetic acid, IAM: indole-3-acetamide, IBA: indole-3-butyric acid, ROS: Reactive Oxygen Species.

Class	Metabolite	Algae	Discussed Effects on Plants	Ref.
Amino acids	D-homocysteic acid, GABA, ornithine, citrulline, hydroxyproline	Chlorophyceae, Phaeophyceae, Rhodophyceae	Nitrogen storage, stress response, osmolytes, pH buffering, antioxidants	[79,80]
Amino acids	Mycosporine-like amino acids	Rhodophyceae	Protection from UV radiation and oxidative damage, osmolytes	[90,91]
Amino acids	Phosphoserine	Phaeophyceae	Phosphoserine-containing peptides lower lipid peroxidation, increase intracellular glutathione and expression of antioxidant enzymes	[82,83]
Amino acid	Proline	Trebouxiophyceae (*Stichococcus*)	Osmolyte, antioxidant, cellular protectant against saline stress	[84]
Amino acid	Taurine	Rhodophyceae (*Porphyra*)	Antioxidant activity	[81,92]
Betains	Glycine betaine, γ-aminobutyric acid betaine and proline betaine	Entire algal kingdom	Osmolytes, ROS scavengers, macromolecules protectans	[28,32,33,89]
Brassinosteroids	Brassinolide, castasterone, typhasterol	Chlorophyceae, Trebouxiophyceae	Promote plant growth, increase crop yield and resistance to biotic and abiotic stresses	[62,93]
Carbohydrate	Trehalose	*Chlamydomonas*, *Chlorella*, *Scytonema*	Osmolyte, carbon reserve, and salt stress protectant	[88]
Carotenoids	E-fucoxanthin	*Nannochloris*, *Tetraselmis*, *Nannochloropsis, Phaedactylium*	Radical scavenger and iron chelator	[70,94]
Flavonoids	Catechin and epicatechin	*Ankistrodesmus*, *Spirogyra*, *Euglena, Caespitella*	ROS scavengers, metal ion chelators, induction of antioxidant enzymes, inhibition of pro-oxidant enzymes	[69]
Phenolic acids	Gallic, syringic, protocatechuic, and chlorogenic acids	*Ankistrodesmus*, *Spirogyra*, *Euglena, Caespitella*	High antioxidant capacity, inhibition of generation as well as scavenging of free radicals, upregulation of antioxidant enzymes	[69]
Phenolic acids	Protocatechuic acid	*Nannochloris*, *Tetraselmis*, *Nannochloropsis, Phaedactylium*	Superoxide anion radical and hydroxyl radical scavenger, metal ion chelator	[70,95]
Phytohormones	Abscissic acid	Entire algal kingdom	Involvement in stress response	[73,74]
Phytohormones	Auxins and cytochinins	Chlorophyceae, Trebouxiophyceae, Ulvophyceae, Charophyceae	Increase of plant growth, yield and defense response against abiotic stress	[63]
Phytohormones	Auxins (IAA, IAM, IBA)	Rhodophyceae, Phaeophyceae, Ulvophyceae	Stimulation of rooting and root growth, increase of resources use efficiency, stress resistance	[34,72]
Phytohormones	Isopentenyladenine, *cis*-zeatin	Chlorophyceae, Phaeophyceae, Rhodophyceae	Stimulation of seed germination, transition between vegetative to generative phases, inhibition of senescence, response to abiotic stresses	[38,96,97]
Phytohormones	Gibberellins	Chlorophyceae, Trebouxiophyceae, Ulvophyceae, Charophyceae	Promote plant growth and resistance to salinity by inducing the degradation of the nuclear family of DELLA TFs and the increase of salicylic acid	[62,98]
Polyamines	Putrescine and spermidine	Chlorophyceae, Charophyceae, Rhodophyceae	Regulation of the cell cycle and increased cell proliferation, stress tolerance	[85,86]
Polyols	Sorbitol and mannitol	*Platymonas*, *Stichococcus*	Osmoprotectans	[88]
Polyphenols	Phloroglucinol, eckol, and dieckol	Phaeophyceae (*Ascophyllum*, *Fucus*)	More efficient ROS detoxification due to the higher number of phenolic rings.	[65,67,68]
Polyphenols	Phlorotannins	Phaeophyceae	Response to both biotic and abiotic stresses, ROS scavenging	[66]
Polysaccharides	Agars, alginates, carrageenans, and fucans	Chlorophyceae, Phaeophyceae, Rhodophyceae	Elicitors of hormonal stress signals (i.e., SA, JA, ethylene) and inductor of resistance to biotic stresses	[77,78]
Tertiary sulphonium compound	3-dimethylsulfoniopropionate	Entire algal kingdom	Osmoprotectant	[32]

**Table 2:** A summary of the biostimulatory actions of algal extracts application on morpho-anatomical, biochemical, and physiological performance of plants under salinity. APX: ascorbate peroxidase, CAT: catalase, GR: glutathione reductase, POD: peroxidase, SOD: superoxide dismutase.

Algae Species	Plant Species	Described Tolerance Mechanisms	Ref.
*Ascophyllum nodosum*	*Agrostis stolonifera*	Increase of SOD activity and resistance to *Sclerotinia homoeocarpa.*	[110]
*Ascophyllum nodosum*	*Amarantus tricolor*	Increased growth parameters (stem length and diameter, root length, number of leaves, fresh and dry weight of leaves, stems and roots).	[111]
*Ascophyllum nodosum*	*Arabidopsis thaliana*	Increase of biomass and polyphenols content.	[112]
*Ascophyllum nodosum*	*Asparagus aethiopicus*	Increase of phenolics, chlorophylls, sugars, proline, anti-oxidant activities, gas exchanges.	[113]
*Ascophyllum nodosum*	*Lactuca sativa*	Increase of weight.	[114]
*Ascophyllum nodosum*	*Paspalum vaginatum*	Increase of ability of plants to maintain higher potassium to sodium ratios.	[115]
*Ascophyllum nodosum*	*Passiflora edulis*	Increase of the initial growth of the seedlings.	[116]
*Ascophyllum nodosum*	*Persea americana*	Increase of plant growth, potassium, and calcium in leaves.	[117]
*Ascophyllum nodosum*	*Solanum lycopersicum*	Increase of beneficial minerals, antioxidants, and essential amino acids.	[108]
*Ascophyllum nodosum*	*Solanum melongena*	Increase of phenols, tannins, total soluble sugars, activity of SOD and APX, and potassium to sodium ratio.	[118]
*Codium taylorii or Pterocladia capillacea*	*Raphanus sativus*	Seed priming induces the synthesis of stress proteins in seedlings under salinity.	[119]
*Dunaliella salina*	*Solanum lycopersicum*	Positive effects on length, dry weight, potassium, potassium to sodium ratio, increase of proline, phenolics, CAT, POD, and SOD activities.	[120]
*Dunaliella salina*	*Triticum aestivum*	Increase of seed germination and seedling growth.	[121]
*Dunaliella spp.*	*Capsicum annuum*	Increase of germination rate, root growth, and reduction of superoxide radical production and lipid peroxidation.	[122]
*Ecklonia maxima*	*Cucurbita pepo*	Increase of yield, shoot biomass, fruit dry matter, total soluble solid contents, chlorophyll content, and photosynthetic rate	[123]
*Fucus spiralis*	*Triticum durum*	Increase of seed germination, growth, and anti-oxidant enzyme activities.	[124]
*Galaxaura obtusata*	*Triticum vulgare*	Increase of SOD activity and shoot length.	[125]
*Grateloupia filicina*	*Oriza sativa*	Increase of proline, SOD, and POD activities, and alleviating salt stress during the seed germination stage.	[126]
*Kappaphycus alverezii*	*Triticum durum*	Increase of tolerance to salt and drought stress.	[36]
*Laurencia obtusa*	*Triticum vulgare*	Decrease of sodium uptake, increase of CAT and SOD activities, carbohydrates, shoot and root length.	[125]
*Lessonia nigrescens*	*Triticum aestivum*	Increase of growth, chlorophyll, and antioxidant activities, decrease of membrane lipid peroxidation, efflux and compartmentation of intracellular ion.	[109]
*Padina pavonica*	*Triticum vulgare*	Increase of CAT and SOD activities, carbohydrates, shoot, and root length.	[125]
*Phaeodactylum spp.*	*Capsicum annuum*	Increase of germination rate, root growth, and reduction of superoxide radical production and lipid peroxidation.	[122]
*Sargassum muticum*	*Triticum vulgare*	Increase of CAT and SOD activities, carbohydrates, shoot and root length.	[125]
*Sargassum muticum Jania rubens*	*Cicer aretinum*	Increase of photosynthetic pigments, soluble sugars, amino acids, phenolics, Na^+^ extrusion SOD, CAT, APX, and POD activity.	[127]
*Scenedesmus obliquus Spirulina platensis*	*Triticum aestivum*	Increase of activities of SOD, APX, CAT, and POD and chlorophyll and carotenoid content.	[128]
*Spirulina maxima Chlorella ellipsoida*	*Triticum aestivum*	Increase of carotenoids, tocopherol, phenolics, proteins, and antioxidant activity	[129]
*Ulva lactuca*	*Triticum aestivum*	Increase of plant growth and yield and antioxidant enzymes activity (SOD, CAT, APX and GR).	[35]
*Ulva rigida*	*Triticum durum*	Increase of leaf pigments, total phenolics, and antioxidant enzymatic activities.	[130]

**Table 3:** A summary of the biostimulatory actions of algal extracts application on molecular mechanisms of plants under salinity. ABA: abscisic acid, AP2: Apetala 2, CK: cytokinins, *DEFL202:* Lam-induced defensin-like 202, LEA: late embryogenesis-abundant; MAP: mitogen-activated protein, MYB: MYB Proto-Oncogene, NHX antiporters: Na+,K+/H+ antiporters, *SnRK2:* SNF1-related protein kinases, *SOS1:* Salt Overly Sensitive 1, WRKY: transcription factors containing the WRKY domain.

Algae Species	Plant Species	Described Tolerance Mechanisms	Ref.
*Ascophyllum nodosum*	*Arabidopsis thaliana*	Downregulation of a putative pectin methylesterase inhibitor, acting as a negative regulator of salt tolerance	[142]
*Ascophyllum nodosum*	*Arabidopsis thaliana*	Upregulation of genes involved in the ABA pathway (*SnRK2*), modification of expression of miRNA and their target genes involved in phosphate homeostasis	[134]
*Ascophyllum nodosum*	*Arabidopsis thaliana*	Response to stress mediated by LEA proteins and dehydrins	[112,131]
*Ascophyllum nodosum*	*Arabidopsis thaliana*	Post-transcriptional and post-translational regulation of Zinc Finger, MYB, and AP2 transcription factors	[131]
*Ascophyllum nodosum*	*Arabidopsis thaliana*	Upregulation of CK and ABA biosynthetic genes	[29]
*Ascophyllum nodosum*	*Asparagus aethiopicus*	Upregulation of stress-related genes involved in the synthesis of proline and chalcones	[113]
*Ascophyllum nodosum*	*Glycine max*	Overexpression of genes involved in ABA catabolism and response to stress	[115]
*Dunaliella salina*	*Solanum lycopersicum*	Upregulation of antioxidant defense and metabolic mechanisms related to jasmonic acid pathway	[120]
*Grateloupia filicina*	*Oriza sativa*	Upregulation of *SOS1* antiporter	[126]
*Kappaphycus alverezii*	*Triticum durum*	Upregulation of stress-responsive genes (e.g., MAP kinase, WRKY transcription factor)	[36]
*Laminaria digitata*	*Arabidopsis thaliana*	Upregulation of genes involved in terpenoid pathway, plastidial-derived secondary metabolite, and *DEFL202*, increasing chloroplast stability	[143]
*Lessonia nigrescens*	*Triticum aestivum*	Upregulation of *SOS1* and *NHX* Na^+^/K^+^ antiporters	[109]
